# Improving Indigenous health equity within the emergency department: a global review of interventions

**DOI:** 10.1007/s43678-024-00687-3

**Published:** 2024-04-29

**Authors:** Tyara Marchand, Kaitlyn Squires, Oluwatomilayo Daodu, Mary E. Brindle

**Affiliations:** 1 Cumming School of Medicine, University of Calgary, Calgary, AB Canada; 2 Department of Surgery, University of Calgary, Calgary, AB Canada; 3 Surgery, Alberta Children’s Hospital, University of Calgary, Calgary, AB Canada; 4 Surgery and Community Health Sciences, Cumming School of Medicine, Calgary, AB Canada

**Keywords:** Indigenous health, Emergency medicine, Quality improvement, Health equity, Santé autochtone, Médecine d’urgence, Amélioration de la qualité, Équité en santé

## Abstract

**Introduction:**

Indigenous health equity interventions situated within emergency care settings remain underexplored, despite their potential to influence patient care satisfaction and empowerment. This study aimed to systematically review and identify Indigenous equity interventions and their outcomes within acute care settings, which can potentially be utilized to improve equity within Canadian healthcare for Indigenous patients.

**Methods:**

A database search was completed of Medline, PubMed, Embase, Google Scholar, Scopus and CINAHL from inception to April 2023. For inclusion in the review, articles were interventional and encompassed program descriptions, evaluations, or theoretical frameworks within acute care settings for Indigenous patients. We evaluated the methodological quality using both the Joanna Briggs Institute checklist and the Ways Tried and True framework.

**Results:**

Our literature search generated 122 publications. 11 articles were selected for full-text review, with five included in the final analysis. Two focusing on Canadian First Nations populations and three on Aboriginal Australians. The main intervention strategies included cultural safety training, integration of Indigenous knowledge into care models, optimizing waiting-room environments, and emphasizing sustainable evaluation methodologies. The quality of the interventions was varied, with the most promising studies including Indigenous perspectives and partnerships with local Indigenous organizations.

**Conclusions:**

Acute care settings, serving as the primary point of access to health care for many Indigenous populations, are well-positioned to implement health equity interventions such as cultural safety training, Indigenous knowledge integration, and optimization of waiting room environments, combined with sustainable evaluation methods. Participatory discussions with Indigenous communities are needed to advance this area of research and determine which interventions are relevant and appropriate for their local context.

## Résumé

**Introduction:**

Les interventions sur l’équité en santé des Autochtones dans les milieux de soins d’urgence demeurent sous-explorées, malgré leur potentiel d’influencer la satisfaction des patients et leur autonomisation. Cette étude visait à examiner et à déterminer systématiquement les interventions en matière d’équité envers les Autochtones et leurs résultats dans les milieux de soins de courte durée, qui pourraient être utilisés pour améliorer l’équité au sein des soins de santé canadiens pour les patients autochtones.

**Méthodes:**

Une recherche dans la base de données a été effectuée de Medline, PubMed, Embase, Google Scholar, Scopus et CINAHL de la création à avril 2023. Pour être inclus dans la revue, les articles étaient interventionnels et comprenaient des descriptions de programmes, des évaluations ou des cadres théoriques dans les milieux de soins de courte durée pour les patients autochtones. Nous avons évalué la qualité méthodologique à l’aide de la liste de contrôle de l’Institut Joanna Briggs et du cadre Ways Tried and True.

**Résultats:**

Notre recherche documentaire a généré 122 publications. 11 articles sélectionnés pour la revue de texte intégral, dont cinq inclus dans l’analyse finale. Deux se concentrent sur les populations des Premières nations canadiennes et trois sur les Australiens autochtones. Les principales stratégies d’intervention comprenaient la formation sur la sécurité culturelle, l’intégration des connaissances autochtones dans les modèles de soins, l’optimisation des environnements des salles d’attente et l’accent mis sur les méthodes d’évaluation durables. La qualité des interventions était variée, avec les études les plus prometteuses, y compris les perspectives autochtones et les partenariats avec les organisations autochtones locales.

**Conclusions:**

Les établissements de soins de courte durée, qui servent de principal point d’accès aux soins de santé pour de nombreuses populations autochtones, sont bien placés pour mettre en œuvre des interventions en matière d’équité en santé, comme la formation en sécurité culturelle, l’intégration des connaissances autochtones, l’optimisation des environnements des salles d’attente, associée à des méthodes d’évaluation durables. Discussions participatives avec Les communautés autochtones sont nécessaires pour faire avancer ce domaine de recherche et déterminer quelles interventions sont pertinentes et appropriées pour leur contexte local.

## Clinician’s capsule

**Table:** 

***What is known about this topic?***
Interventions focused on improving health equity for Indigenous patients have been proven to be successful in primary care settings, interventions within the realm of emergency care have not been well studied and are an important target area to improve Indigenous health experience within Canada’s emergency departments (ED).
***What does this study ask?***
What interventions are working to address Indigenous health equity within emergency care across the globe?
***What did this study find?***
There are four key aspects to consider when implementing Indigenous health equity interventions within emergency care, including: staff cultural safety education, designing welcoming waiting rooms, integration of Indigenous models of care, and long-term evaluation methods inclusive of Indigenous perspectives.
***Why does this study matter to clinicians?***
Indigenous populations are the youngest and fastest-growing population in Canada and have disproportionate ED visit rates when compared to their non-Indigenous counterparts. Emergency physicians and staff should be aware of the potential ways that their institutions can advance health equity for Indigenous patients.

## Background

In Canada, and other European-colonized countries, Indigenous people experience significant disparities in health outcomes and healthcare access [1, 2]. Inequities persist across measures of health quality such as mortality, suicidality, and chronic and infectious disease burdens [3, 4]. Health inequities for Indigenous people are interactional and multifactorial and are perpetuated by the longstanding impacts of colonialism, anti-Indigenous racism, structural and systemic barriers, and the colonial foundation of Western healthcare [5, 6]. There have been notable efforts to effect positive change including advancements in cultural safety programming [7], increasing Indigenous-led research and prioritization of Indigenous-led healthcare initiatives [8].

Emergency care is a critical health access point for many Indigenous peoples and presents an opportunity for further positive health impact [9]. Indigenous patients tend to have higher rates of emergency department (ED) visits [10], decreased length of ED stays [11, 12], higher hospital admission rates [13], increased disease complexities [14, 15] and incorrectly assigned lower triage scores [9, 13]. The compounding factors increasing ED utilization include inadequate access to routine primary care, increased propensity for acute pathologies [15], and healthcare avoidance due to the significant anti-Indigenous biases present in our health system [16, 17]. Considering these factors, EDs can be areas of health equity reform, providing an opportunity to increase the quality of Indigenous healthcare.

This review examines health equity interventions within acute care settings across four countries with Indigenous minorities that share a history of colonialism [2]; Canada, United States, Australia, and New Zealand. This project attempts to answer the following questions:

1. What emergency care equity projects have occurred that specifically target Indigenous populations?
2. How did these projects address health disparities for Indigenous patients?

## Methods

### Search strategy

This review was conducted according to PRISMA guidelines [18, 19], and investigates the landscape of Indigenous equity interventions within acute care settings located in countries with original Indigenous inhabitants including Canada, United States, Australia, and New Zealand.

**Fig. 1:**
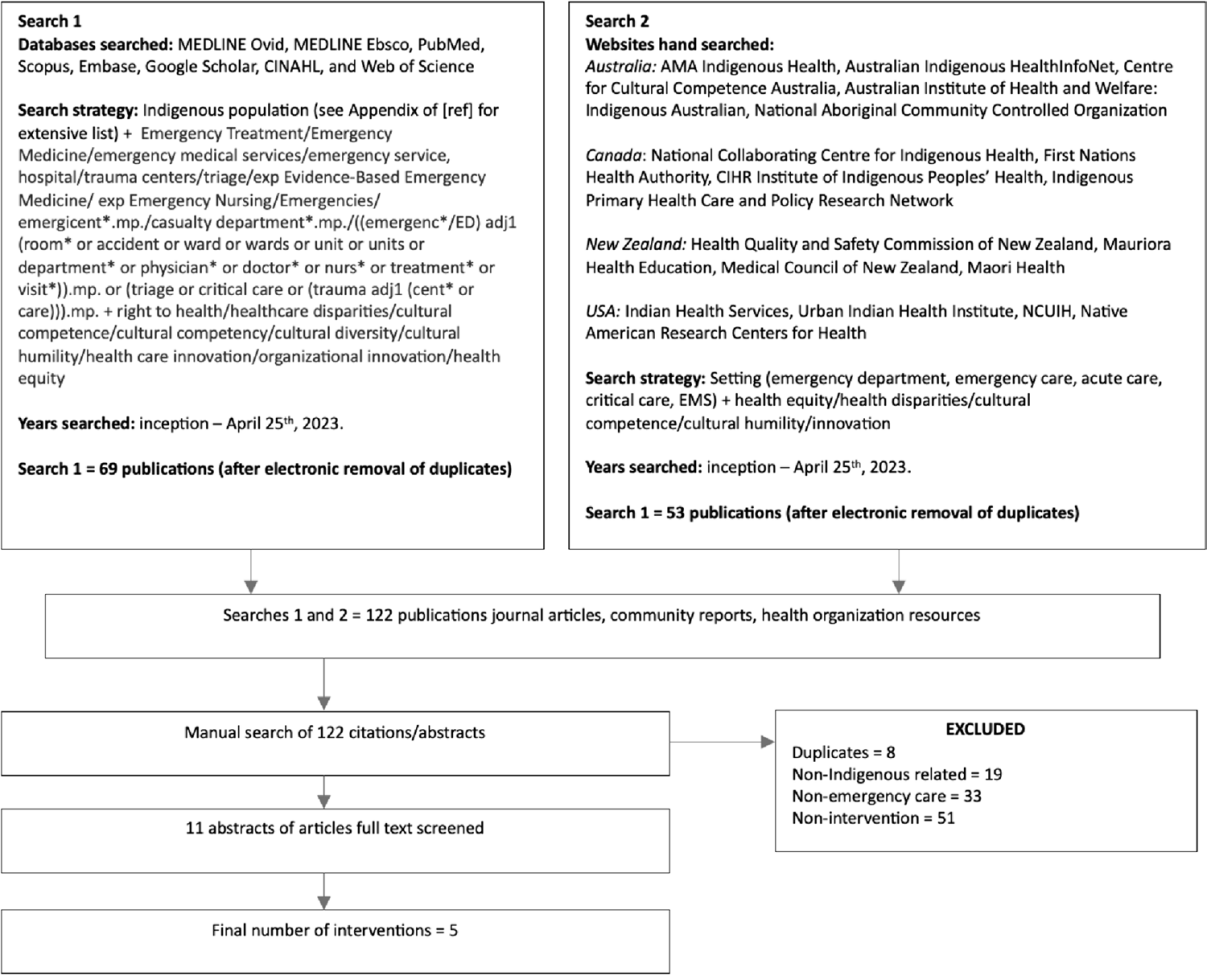
PRISMA diagram with search strategy

The search strategy was developed by a health systems analyst and reviewed with two Indigenous health scholars. The study protocol has been published [20]. The detailed search strategy is summarized in Fig. 1 below. The search included all citations from inception until April 2023.

### Eligibility criteria

The PICO statement for this study is in Table 1. This review examined emergency care in countries with Indigenous minority populations, history of genocide and settler colonization, and comparable economic and social structures. These countries include Australia, Canada, New Zealand and the United States. Included studies had to be intervention-based and aligned with health equity concepts or integrated Indigenous ways of knowing, doing, and being. For this review, Indigenous health equity refers to the concept of fairness and justice within health systems, specifically to the capacity of institutions to deliver non-discriminatory health care to Indigenous patients [21, 22].

**Table 1:** PICO statement

Population	Indigenous populations accessing acute care services in Australia, Canada, New Zealand, and the United States
Intervention	Interventions focused on specifically addressing Indigenous health equity within emergency care settings
Comparison	N/A
Outcomes	Primary outcomes include intervention evaluation, process of care measurements, and patient satisfaction scores
Study design	Primary empirical studies, theoretical studies, implementation studies

This inclusion criteria encompasses department-wide initiatives, emergency medicine training programs, staff education programs, and initiatives that target health structure inequities experienced by Indigenous populations. A comparison group was not required. All outcomes were included.

### Relevance screening

Citations were imported into Covidence, where duplicates were removed. Screening was conducted in two stages. First, titles and abstracts were screened, followed by a screening of full texts. Screening was performed independently by two reviewers (TM, KS). Disagreements were resolved by consensus or supervisor input. Reviewers met regularly to address any conflicts.

### Data extraction and analysis

The data extraction criteria were derived from the Cochrane Collaboration Handbook for Systematic Reviews of Health Promotion and Public Health Interventions [19] and included the intervention(s) being evaluated, sample characteristics (including population, sample size, literature type), study design, outcomes assessed, and observed effects.

### Methodological quality

The methodological quality of included studies was assessed using both a non-Indigenous and Indigenous methodology framework. This study utilized the Joanna Briggs Institute checklist for systematic reviews, (Table 2) [23]. The ways tried and true (WTT) framework was invoked as a quality scale to assess how interventions aligned with Indigenous epistemologies (Table 3) [24]. WTT emphasizes the importance of Indigenous culture, community engagement and collaboration, and assists in determining if interventions reflect Indigenous aspirations and priorities. This combined approach facilitated the integration of perspectives and methodologies from both Indigenous and Western contexts.

**Table 2:** Joanna Briggs Institute (JBI) critical appraisal checklist for qualitative research

Study first author, year	Criteria										
	Consistency between theoretical framework and methods?	Consistency between methods and objectives?	Consistency between methods and data collection?	Consistency between methods and data analysis?	Consistency between methods and interpretation of results?	Inclusion of the researcher’s cultural or theoretical location?	Inclusion of the influences between the research and the researcher?	Adequate representation of the participants?	Inclusion of ethical approval by an appropriate body?	Adequate flow from interpretation to conclusion?	Overall appraisal
Barnes, 2022 [16]	Yes	Yes	Yes	Yes	Yes	Unclear	Yes	No	Unclear	Yes	Include
Gadsden, 2019 [25]	Yes	Yes	Yes	Yes	Yes	No	Unclear	Yes	Yes	Yes	Include
Preisz, 2022 [26]	Yes	Yes	Yes	Yes	Yes	No	No	No	Unclear	Yes	Include
Carter, 2021 [27]	Yes	Yes	Yes	Yes	Yes	Yes	Yes	Yes	Yes	Yes	Include
Varcoe, 2022 [28]	Yes	Yes	Yes	Yes	Yes	No	No	No	Yes	Yes	Include

**Table 3:** Ways tried and true framework for public health interventions

Study first author, year	Criteria (score out of 5 and rationale)					
	Community-based	Wholistic	Integration of Indigenous cultural knowledge	Building on community strengths and needs	Partnership and collaboration	Effectiveness
Barnes, 2022 [16]	Intervention based on need identified, includes structures for involvement of community, but not led by; 2	Intervention addresses a couple dimensions, but limited cross sector and implementation environments, view of wellness, and involvement of community; 2	Indigenous knowledge informs the intervention with participatory processes, but not all aspects; 3	Intervention design acknowledges and builds on strengths of the community and attempts to fill gaps; 3	Intervention involves partners guided by the developed framework but room for improvement for increased collaboration; 3	Emerging data suggests positive outcomes, but reporting is preliminary; 1
Gadsden, 2019 [25]	Intervention based on need identified, includes community in all aspects, but not rooted in systems of community; 3	Intervention addresses multiple dimensions and environments/group but is limited in its view of wellness; 3	Indigenous knowledge is used in the ED projects and informs the QI program, but the QI program is not fully integrated in; 2	Intervention design acknowledges and builds on strengths of the community and attempts to fill gaps; 3	Intervention involves partners guided by the developed framework but room for improvement for increased collaboration; 3	For accurate recording 6/8 EDs had significant increases, but there was no significant trend found in addressing incomplete ED visits; 2
Preisz, 2022 [26]	Intervention based on need identified, rooted mainly in systems of community, but not owned by; 3	Intervention is wholistic and targets multiple environments and groups, and implements a wholistic view of health; 4	Values, culture, and perspectives of the community are integrated into and inform all aspects of the intervention, from planning through to implementation; 4	Intervention design acknowledges and builds on strengths of the community and attempts to fill gaps; 3	Intervention involves partners guided by the developed framework; but room for improvement for increased collaboration; 3	Significant improvements to quality and equity of access to medical care for Aboriginal patients, well received by staff and patients, with no adverse effects on delivery of services to other patient groups; 3
Carter, 2021 [27]	Intervention from within community to address need, rooted mainly in systems of community, and owned by the community; 4	Intervention is wholistic and implements a wholistic view of health, but limited in targeted environments and groups due to scale of intervention; 3	Values, culture, and perspectives of the community are integrated into and inform all aspects of the intervention, from planning through to implementation; 4	Intervention design acknowledges and builds on strengths of the community and attempts to fill gaps; 3	Intervention involves partners guided by the developed framework; but room for improvement for increased collaboration; 3	Emerging data suggests positive outcomes, but reporting is limited due to small scale of project; 1
Varcoe, 2022 [28]	Intervention idea comes from outside of the community and is implemented with limited community involvement; 1	Intervention addresses a couple dimensions and involves multiple cross sector and implementation environments, but has a limited view of wellness and involvement of community; 2	Values, knowledge, culture and community perspectives play an informal role in the intervention; 1	Intervention shows informal acknowledgement of community strengths and needs (gaps). Capacity may be being built, but not among First Nations, Inuit or Métis peoples within the community; 1	There are some collaborative partnerships associated with the intervention, but there have been substantial challenges in implementing the plans or involving partners (e.g., pandemic); 1	Emerging data suggests positive outcomes, but reporting is preliminary; 1

## Results

Throughout the screening process, Cohen’s kappa consistently exceeded the 0.80 threshold [29]. Five studies were identified after the screening. Table 4 summarizes the characteristics of intervention evaluations. Multiple outcomes were examined. No study evaluated costs.

**Table 4:** Characteristics of evaluations of health equity interventions

First author, year, publication type	Country, location, site	Target population	Theoretical framework	Research design	Intervention type	Intervention level	Intervention evaluation	Process of care measurements
Barnes, 2022, journal article [16]	Australia, Melbourne, tertiary ED	Aboriginal and Torres Strait Islander peoples	Principles of participatory action research and appreciative enquiry	Creation of theoretical framework and working group	Education	Organizational	Development of initiatives for cultural safety and trauma-informed care education	Action areas identified: relationships, education, welcoming waiting room, accurate identification
Gadsden, 2019, journal article [25]	Australia, New South Wales, 8 EDs in rural and urban areas	Aboriginal and Torres Strait Islander peoples	Continuous Quality Improvement framework	Evaluation of Aboriginal identification in hospitals	Evaluation	Regional	Proportion of patients correctly identified in ED systems; rates of incomplete ED visits; effect on cultural appropriateness of ED environments	Significant increase in accurate recording of status (6/8 ED). No decrease in incomplete ED visit trend
Preisz, 2022, journal article [26]	Australia, Sydney, major tertiary ED	Aboriginal and Torres Strait Islander peoples	Yerin Dilly' bag model and Clinical yarning	Implementation and evaluation of a new model of care	Model of care	Organizational	Rates of incomplete treatment, “left at own risk”, and “did not wait”	Incomplete treatment rate 5 × lower DNW and LAOR significantly decreased (pre-intervention: 19.5% of patients, post-intervention: 1.6%)
Carter, 2021, journal article [27]	Canada, rural northern British Columbia	First Nations peoples	Critical paradigm, post-colonial theories of Fanon, Foucault, and Kristiva; and Grounded Theory	Dialogue to inform culturally safe practices in ED	Education	Organizational	Qualitative inquiry and interpretive thematic analysis	Key themes: key attributes of culturally safe and unsafe care, current culturally safe practices, factors that challenge cultural safety, suggestions to enhance cultural safety
Varcoe, 2022, journal article [28]	Canada, British Columbia, 3 EDs in urban, suburban, and rural areas	ED patients	Equity-oriented health care (EHOC), complexity theory	Evaluation of EQUIP intervention at 3 diverse EDs in BC	Evaluation	Organizational	Patient demographics, experiences of discrimination, and overall ratings of care	Increased intervention activities related to significantly higher patient perceptions of quality of care and significant decrease in leaving without care rate

### Target population

Three studies examined interventions involving Aboriginal and Torres Strait Islander peoples from Australia [16, 25, 26] and two First Nations populations in Canada [27, 28]. No interventions were identified involving Indigenous populations within the US or New Zealand.

### Theoretical frameworks

Each study had differing frameworks grounding their respective interventions. One study was rooted in Indigenous knowledge and pedagogy using the Yerin Dilly Bag Model and yarning [26]. The Yerin Dilly Bag Model [26] is an approach to Indigenous health research engagement through metaphorically “packing” and “carrying” core values and actively resisting colonialist/Western individualistic approaches [30]. Yarning [26], a tool recognized by Indigenous Australians, creates a culturally safe space for meaningful conversations rooted in Indigenous pedagogy [30].

Three of the studies’ focused on cultural safety [26–28]. Critical paradigm, post-colonial theories, and grounded theory were the frameworks used in the study by Carter et al. [27]. These explored cultural safety from a critical lens focused on the impacts of colonization and its continuous effects [27, 31, 32]. Equity-oriented health care and complexity theory were used in the Varcoe et al. study; [28] both grounded in cultural safety, harm reduction, trauma- and violence-informed care to improve equity at an organizational level [28].

Two studies were rooted in quality improvement (QI) and cyclical refinement frameworks [16, 25]. Principles of participatory action research and appreciative enquiry are frameworks for engagement and participant buy-in for social change on an organization/community-level [16]. Continuous Quality Improvement is a system of cyclical reflection and refinement to improve organizational processes and outcomes [25]. While not rooted in Indigenous pedagogy, it has begun to show promise within Indigenous-focused healthcare improvement studies [25, 33].

### Intervention strategies

The main intervention strategies employed included: QI evaluation [25, 28], model of care [26], and education programs [16, 27]. All of which were conducted on an organizational level, except for the Gasden et al. study [25] which took place regionally.

### Sustainable evaluation methodologies

Both studies performing QI evaluation focused on cultural safety and equity of Indigenous peoples within multiple EDs. The first looked at Indigenous identification in hospitals [25]. Each ED implemented a QI project focusing on working with Indigenous people that followed common objectives: encourage Indigenous self-identification, improve staff cultural competence, collaborate with Indigenous organizations, and reduce incomplete ED visits among Indigenous patients. The second focused on ED interventions aiming to promote equity for Indigenous people at an organizational level through collaboration among health researchers, hospital staff, and Indigenous/community leaders [28]. In both studies, research teams provided QI frameworks, working groups, and support for ED programs [25, 28].

### Integrated care models

One study implemented and evaluated a new model of care [26] co-designed with the hospital’s Aboriginal Health Unit [26]. An adaptive model for Indigenous ED patients was created with the objective of developing a person-centered approach to improve care, connections, cultural competency, and both verbal and non-verbal communication [26]. Specifics of the model of care include direct referral after triage, ability to leave/return at any point, prioritized medication dispensing, and assigned Aboriginal healthcare worker and senior clinician [26].

Four papers discussed the importance of integrating Indigenous ways of knowing, doing and being into healthcare [16, 26–28]. Potential strategies include prioritizing Indigenous epistemologies, such as meeting patients where they are, flexible understanding of time, and focusing on relationship building between staff, patients, and their communities.

### Environment optimization

Varcoe et al. [28] used strategies to create an equitable waiting room at an urban ED with culturally appropriate triage signage, equity-oriented television messaging, and partnered with local artists to introduce Indigenous artwork in the waiting room. Follow-up evaluation for these interventions showed an overall decrease in patients who leave without being seen. Carter et al. [27] discussed the importance of having a safe and open environment in the ED through open communication methods, explaining triage processes and waiting rooms, and having accessible patient navigators.

### Cultural safety education

Two studies employed education interventions: one through creating a theoretical framework and working group [16], the other through a dialogue to inform culturally safe practices in the ED [27]. The theoretical framework and working group, led by an ED consultant and collaboration with the hospital’s Aboriginal Health Unit, enabled critical self-reflection, constructive discourse, and innovation [16]. The dialogue hosted in the other study was guided by a 7-member advisory committee, with four Indigenous members, with the purpose of learning about and disrupting pre-existing power imbalances [27]. This article discussed the importance of cultural safety training and how education and compassionate communication play a pivotal role in high-quality Indigenous emergency care. Key aspects of cultural safety training were discussed, including understanding, and acknowledging historical traumas perpetrated on Indigenous peoples, recognizing that the ED is often the only avenue of care for Indigenous patients, and confirming the importance of invoking a trauma-informed care approach when working with Indigenous patients [27].

### Methodology

Two studies employed qualitative methodologies [16, 27], one quantitative [26], and two mixed-method [25, 28]. The qualitative methodologies involved a participatory action approach alongside an interpretive thematic analysis [16, 27]. The quantitative study used ED system data to understand “left at own risk” (LAOR) and “did not wait” (DNW) rates for Indigenous patients [26]. One mixed-method study used qualitative inquiry through various tools, including: sharing circles, interviews, self-assessment questionnaire, wordle, poem, and ethnographic tools, combined with multiple baseline design and secondary review of ED Indigenous identification data [25]. The other conducted surveys to gather data on patient demographic characteristics, experiences of ED discrimination, and overall care ratings [28].

### Intervention effectiveness

Due to the early nature of the interventions, effectiveness data was limited. However, there were important observations that can guide future work. Education intervention studies highlighted key themes and action areas: culturally safe care practices, staff cultural competency, relationships, education, and communication [16, 27]. One of the evaluation studies reported a significant increase in accurate recording of Aboriginal status in six of eight EDs, but no statistically significant decrease in incomplete visits [25]. The other study found that increased sustained intervention activities were related to higher patient perceptions of care quality and a significant decrease in leaving without care [28]. The model of care intervention reported a 5 times lower incomplete treatment rate and significantly decreased “left at own risk” and “did not wait” rates [26].

## Discussion

### Interpretation of findings

This study uncovered four key priority areas: effective evaluation strategies, staff cultural safety education, engaging in locally relevant care models and optimized healthcare environments. Effective evaluation adopts a two-eyed seeing approach, recognizing the importance of incorporating Indigenous perspectives alongside Western methodologies. The pursuit of Indigenous health equity goes beyond relying solely on isolated data points. Instead, it requires considering the broader context and acknowledging that collaborative efforts between health organizations and Indigenous communities can contribute to advancing health equity. Additionally, education is crucial for Indigenous health equity and Indigenous-led efforts incorporating a trauma-informed approach has been proven to be exceptionally impactful and transformative. In alignment, the integration of Indigenous ways of knowing into Western healthcare models have demonstrated improvements in Indigenous health outcomes [34]. Potential ways to achieve this are Indigenous patient navigators, collaboration with Indigenous communities and organizations, creating space for traditional healing practices, and respect for Indigenous values. Building on this knowledge integration, creating a welcoming environment for patients in the ED is paramount and has been linked to decreased ED left without being seen rates, patient empowerment, and overall satisfaction with care [28]. Interventions occurred in Australia and Canada, potentially influenced by well-established Indigenous-led healthcare systems within larger westernized and academic frameworks. This contrasts with New Zealand’s culturally entrenched parallel system and the early stages of Indigenous healthcare autonomy in the US.

### Comparison to previous studies

To date, there has not been a comprehensive review of the literature attempting to identify Indigenous health equity interventions within the ED. Nevertheless, existing publications offer a valuable starting point for improving person-centered care for Indigenous populations and serve as a foundation for future research. Within the primary care sphere, when Indigenous patients engage with their culture and include traditional methods in their care, they report being more empowered and satisfied with their healthcare experience [35]. By acknowledging and incorporating traditional knowledge and cultural norms, EDs and health systems can better meet the needs of Indigenous patients. Often, studies that explore Indigenous health equity lack comprehensive and long-term evaluation. These challenges can be partially attributed to the difficulty of accessing Indigenous participants [36] and the substantial time and resources required to create ethically based, long-term and collaboration-driven projects. Our review re-solidified that evaluation methodologies should prioritize Indigenous perspectives throughout the entire research process, from idea conception to project implementation.

Prior research and anti-oppression training within the Indigenous health sphere has been heavily focused on cultural safety training as a single intervention with limited impact [28, 37]. While educating staff is an integral part of Indigenous health equity, this alone is inadequate as a single intervention and broader system-level interventions must concomitantly be introduced. Additionally, cultural safety training has been shown to be most effective when done in partnership with Indigenous peoples and grounded within Indigenous epistemologies [37, 38]. Combining education with broader systemic changes, such as the integration of culture within healthcare, will be pivotal in making substantial progress towards achieving Indigenous health equity.

### Strengths and limitations

We cannot draw broad conclusions from the limited number of identified studies, nor can we assess the effectiveness of unpublished interventions. We also acknowledge that emergency medicine is not intentionally structured to provide longitudinal care or address all dimensions of wellness for Indigenous patients. Acute care services hold a significant position in addressing the health needs of Indigenous peoples, who often use the ED as a primary point of healthcare access [39]. This review is focused on an Indigenous context, potentially inadvertently excluding equity projects targeting the larger BIPOC community, however, lessons learned may be applicable to other BIPOC initiatives.

To prioritize Indigenous-led initiatives, we utilized the WTT framework for scoring interventions, recognizing that non-Indigenous-led projects may not be entirely representative of a community’s priorities. In future project implementation, we plan to engage in dialogues with Indigenous communities to ensure local relevance. Finally, Indigenous health initiatives may be community or Indigenous health-organization led, which may lead to variations in reporting and documentation. We made efforts to survey relevant Indigenous health websites, but some interventions may not have been captured in this review.

### Research and clinical implications

This review exposes the scarcity of research exploring Indigenous health equity in emergency care, and the critical need for research on equity-focused interventions. Impactful interventions should be tailored to meet community needs that are locally relevant and sustainable. We aim to leverage this work to inform interventions within our local EDs and advocate to policymakers the need to invest in Indigenous health equity within Canada’s ED’s. Ultimately, we aim to enhance knowledge around Indigenous health in Canada and the importance of greater equity for Indigenous peoples and their communities.

## Conclusion

Intervention strategies for Indigenous health equity in emergency care include QI evaluation, model of care implementation, and educational programs at the organizational and regional level. These interventions aim to promote cultural safety, improve cultural competence, and enhance collaboration with Indigenous peoples. To effectively implement these components, organizations must adopt a collaboration-driven, two-eyed seeing approach, engage in sustainability with clear evaluation strategies, and tailor initiatives to the needs of their local Indigenous populations. Individually, providers can improve health equity through participation in cultural safety training, encouraging their department to implement Indigenous health equity strategies, and supporting Indigenous-led initiatives.

## Data Availability

The data is available from the authors upon reasonable request.
